# Metformin protects retinal ganglion cells in a preclinical model of retinal ischemia/reperfusion injury and stabilizes visual field in diabetic patients with glaucoma

**DOI:** 10.1038/s41420-025-02824-y

**Published:** 2025-11-24

**Authors:** Andrea Satriano, Alessio Martucci, Annagrazia Adornetto, Elisabetta Benfatto, Gianluca Tettamanti, Daniele Bruno, Maria Tiziana Corasaniti, Giacinto Bagetta, Carlo Nucci, Rossella Russo

**Affiliations:** 1 Department of Pharmacy, Health and Nutritional Sciences, Section of Preclinical and Translational Pharmacology, University of Calabria, Arcavacata di Rende, Italy; 2 Department of Experimental Medicine, Ophthalmology Unit, University of Rome “Tor Vergata”, Rome, Italy; 3 Department of Biotechnology and Life Sciences, University of Insubria, Varese, Italy; 4 Department of Health Sciences, University “Magna Graecia” of Catanzaro, Catanzaro, Italy

**Keywords:** Glaucoma, Cell death in the nervous system

## Abstract

Metformin, a first-line treatment for type 2 diabetes, has gained attention as a promising neuroprotective agent due to its pleiotropic effects - including anti-inflammatory, anti-apoptotic, and autophagy-enhancing properties. Here we provide both preclinical and clinical evidence demonstrating the neuroprotective effects of metformin in the context of retinal ganglion cell (RGC) degeneration, a hallmark of glaucoma, a leading cause of irreversible blindness for which no direct RGC-neuroprotective therapies are currently available. In a mouse model of retinal ischemia/reperfusion injury systemic administration of metformin significantly prevented RGC loss and preserved retinal structure. Enhanced phosphorylation of AMP-activated protein kinase (AMPK) was observed, along with increased autophagosome formation and upregulation of key mitophagy markers - including LC3II, optineurin, and Parkin - indicating improved mitochondrial quality control mechanisms. Proteomic analysis revealed that metformin modulated several proteins implicated in mitochondrial respiratory function, ubiquitination, and intracellular trafficking, suggesting broader effects on retinal cellular homeostasis. Complementing our preclinical observations, a retrospective clinical study in diabetic patients with glaucoma showed that individuals treated with metformin maintained stable visual field (VF) parameters over a six-month period, whereas those treated with insulin exhibited significant VF deterioration. These findings position metformin as a promising intraocular pressure (IOP)-independent neurotherapeutic for slowing or preventing glaucomatous neurodegeneration.

## Introduction

Retinal ganglion cells (RGCs) are the primary projection neurons of the retina, responsible for transmitting visual information to the brain via their axons, which converge to form the optic nerve [1]. This process is essential for vision, and RGC dysfunction or loss is a hallmark of both traumatic and degenerative ocular diseases, most notably diabetic retinopathy and glaucoma [2].

Glaucoma is a multifactorial optic neurodegenerative disease and the leading cause of irreversible blindness worldwide with prevalence expected to reach approximately 111.8 million individuals by 2040 [3]. Despite advances in the understanding of glaucoma pathophysiology and its therapeutic management, no definitive treatments are currently available to halt or reverse RGC degeneration. Existing strategies focus on lowering intraocular pressure (IOP), a major risk factor alongside aging, in order to delay the progression of glaucoma. However, while ocular hypotensive therapies have been clinically proven to help preserve the visual field (VF) [4, 5], a significant proportion of patients continues to experience disease progression despite well-controlled IOP [6]. This underscores the urgent need for IOP-independent neuroprotective therapies that can sustain and enhance RGC function and prevent their apoptotic death [7–9].

Metformin, a synthetic biguanide derivative introduced in the late 1950s, remains the first-line treatment for newly diagnosed type 2 diabetes [10]. As a glucose-lowering agent, metformin improves insulin sensitivity, inhibits hepatic gluconeogenesis, and reduces intestinal glucose absorption [11]. While the mechanisms underlying these effects are still partially unraveled, most converge on the activation of the ubiquitous adenosine monophosphate (AMP) protein kinase (AMPK), a key sensor of cellular energy status and regulator of metabolic homeostasis [12].

In recent decades, metformin has garnered increasing interest for its pleiotropic benefits beyond glycaemic control, showing potential in several conditions such as cancer, cardiovascular diseases, and neurodegeneration [13–15]. These effects are ascribed to its anti-inflammatory, anti-apoptotic, anti-angiogenic, and anti-aging properties [16, 17].

Of particular relevance to neurodegenerative diseases, metformin has been shown to modulate autophagy [18, 19] and support mitochondrial function, improving mitochondrial quality control, enhancing fission, and reducing oxidative stress [18, 20].

Previous studies have demonstrated that sustaining autophagic activity - through pharmacological or genetic approaches - confers neuroprotection to RGCs exposed to glaucoma-related stressors [21–24]. In a model of retinal ischemia/reperfusion injury, our group showed that inducing autophagy, either via the mTOR (mammalian target of rapamycin) inhibitor rapamycin or by starvation, significantly improved RGC survival [23, 25]. However, chronic mTOR inhibition or severe dietary restrictions is unlikely to be clinically viable due to adverse effects and poor patient compliance.

Given its favorable safety profile, along with clinical evidence linking its use in diabetic patients to a reduced risk of open-angle glaucoma [26–28], metformin is a compelling candidate as disease-modifying agent in glaucoma. Therefore, the aim of our study is to investigate the neuroprotective potential of metformin in a preclinical mouse model of retinal ischemia/reperfusion injury, and in a clinical cohort of diabetic patients with glaucoma.

## Results

### Systemic administration of metformin prevented RGC loss induced by ischemia/reperfusion and preserved retina morphology

Retinal ischemia induces significant RGC loss [23, 25]. The animal model used in this study is characterized by a mechanical, pressure dependent occlusion of the central retinal artery, resulting in ischemia followed by reperfusion upon pressure release. This ischemia-reperfusion insult mimics some aspects of glaucomatous optic neuropathy, especially in patients with vascular dysregulation or impaired autoregulation of ocular blood flow. In this context, the model is useful for testing neuroprotective strategies that may mitigate RGC death regardless of IOP control, which is highly relevant for glaucoma patients whose disease progresses despite normalized IOP [2, 29, 30].

**Fig. 1:**
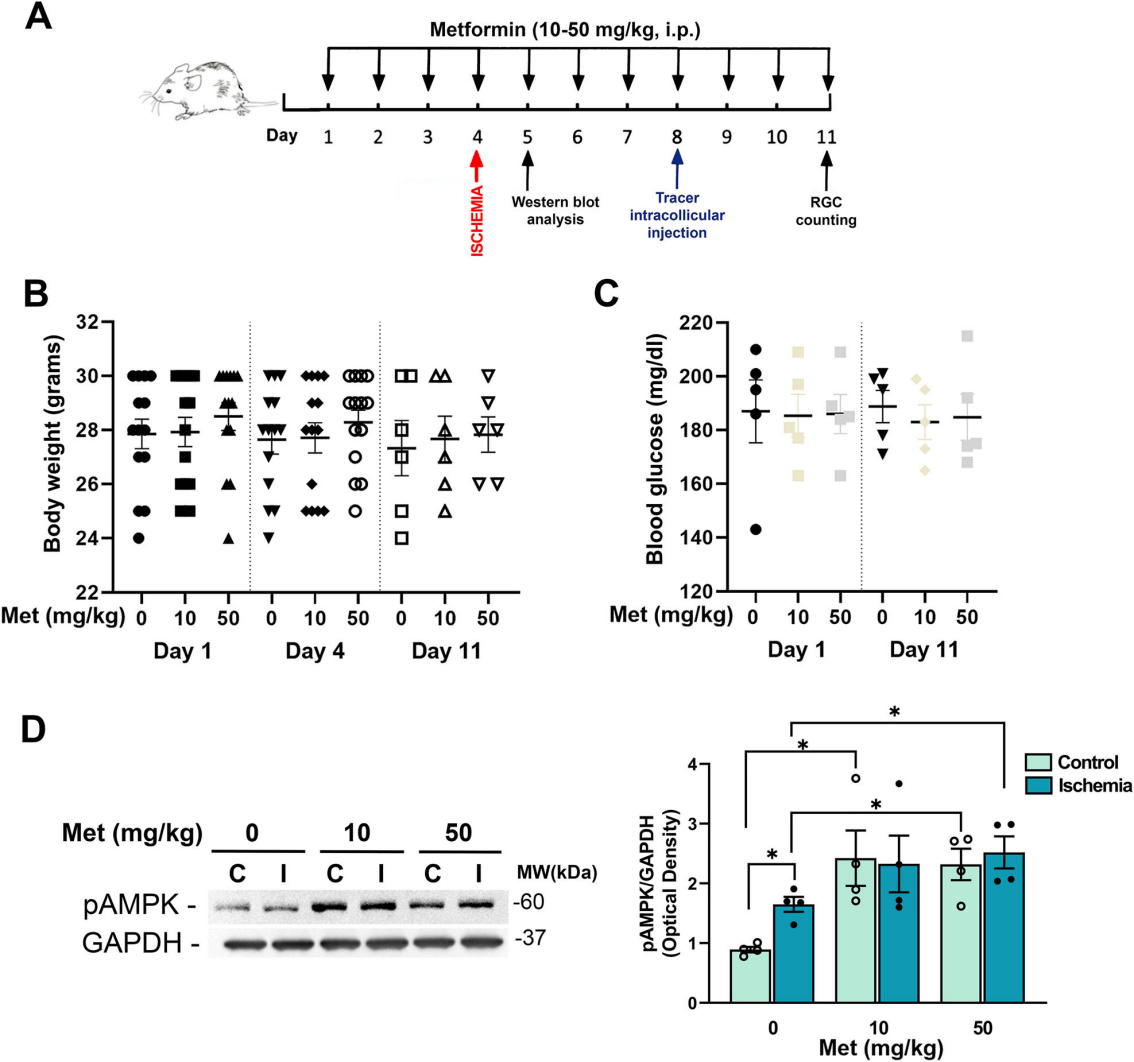
Effect of metformin on body weight, blood glucose level and AMPK phosphorylation in mice subjected to retinal ischemia/reperfusion injury. **A** Metformin treatment schedule. Metformin (10 and 50 mg/kg) or vehicle was administered intraperitoneally (i.p.) once daily for 11 consecutive days; retinal ischemia (I) was induced on day 4. Mice were sacrificed 24 h post-ischemia (24 h reperfusion) for biochemical analysis or after 7 days for the evaluation of RGC survival. Body weight (**B**) and blood glucose levels (**C**) in metformin- and vehicle treated mice over the treatment period. No significant changes were detected. **D** Representative immunoblot showing AMPK phosphorylation (Thr172) in retinas from metformin- (10, 50 mg/kg) or vehicle-treated (0) mice subjected to unilateral ischemia (I). Contralateral eye served as control (**C**). Histograms report the densitometric analysis of the bands normalized to internal loading control (GAPDH). Data are reported as mean ± s.e.m. (*n* = 4). *p < 0.05 (Met metformin, MW molecular weight).

To evaluate the neuroprotective effect of metformin, mice were systemically treated for 11 consecutive days (10 and 50 mg/kg/day) [31, 32] and retinal ischemia was induced at the fourth day of treatment (Fig. 1A).

Body weight was daily monitored and glycemia was checked on the first and last day of the treatment period with no significant differences observed between metformin and vehicle treated animals (Fig. 1B, C).

Under this administration schedule, metformin reached effective concentrations in the retina as shown by the increased phosphorylation of AMPK, a well-characterized downstream target of the drug [12, 33, 34].

Metformin 10 or 50 mg/kg enhanced AMPK phosphorylation (Thr172) in control retinas and abrogated the difference in AMPK phosphorylation level between control and ischemic retinas observed in the vehicle treated group (Fig. 1D). Notably, only the 50 mg/kg dose significantly enhanced AMPK phosphorylation in ischemic retinas (Fig. 1D)

**Fig. 2:**
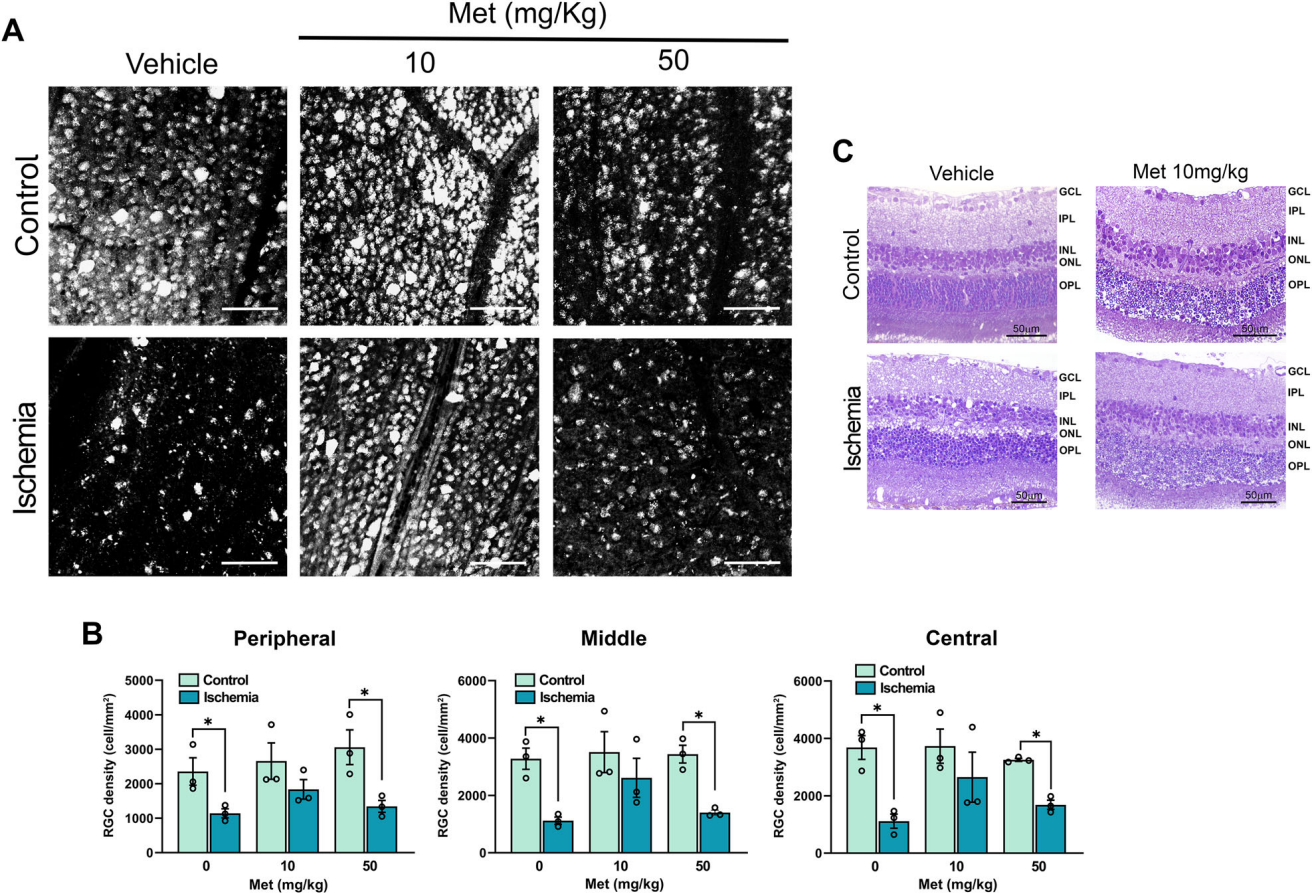
Metformin prevents RGC loss induced by retinal ischemia/reperfusion injury and preserves retinal morphology. **A** Representative fluorescent photomicrographs of FluoroGold-labeled RGCs in mid-peripheral retina from control (C) and ischemic (I) retinas of vehicle- or metformin-treated mice. Scale bar 75 μm. **B** Quantitative analysis of RGC density in peripheral, middle and central retina from metformin- (10, 50 mg/kg) or vehicle-treated mice. Ischemia was induced in the right eye (I); left eye was used as control (C). Results are reported as mean ± s.e.m. (*n* = 3). *p < 0.05. **C** Representative hematoxilin and eosin-stained retinal sections from mice treated with metformin 10 mg/kg or vehicle. GCL ganglion cell layer, IPL inner plexiform layer, INL inner nuclear layer, ONL outer nuclear layer, OPL outer plexiform layer. (Met metformin).

Seven days after ischemia, RGC density was significantly preserved across the peripheral, middle and central retinal regions in mice treated with metformin 10 mg/kg, whereas the 50 mg/kg dose was ineffective (Fig. 2A, B).

Histological evaluation revealed that retinas exposed to ischemia injury from mice treated with metformin 10 mg/kg displayed preserved laminar organization, ganglion cell layer (GCL) density, and fewer structural disruptions compared to vehicle-treated animals (Fig. 2C).

### Ultrastructural features of autophagy were observed in retinas from metformin-treated mice subjected to ischemia/reperfusion injury

Metformin is recognized as a caloric-restriction mimetic and autophagy modulator [19, 35, 36].

To detect changes in autophagic structures, transmission electron microscopy (TEM) - the gold standard for autophagosome detection- was performed [37].

**Fig. 3:**
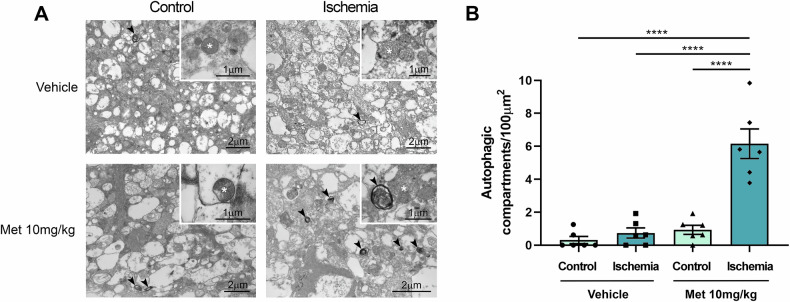
Ultrastructural analysis of retinal tissue in metformin-treated mice after ischemia/reperfusion injury. **A** Representative TEM images of the cytoplasmic content in cells located in the GCL from vehicle- or metformin-treated (10 mg/kg) mice 24 h post-ischemia. **B** Quantification of autophagic compartments in the cytoplasm of neurons from control and ischemic retinas treated with vehicle or metformin (10 mg/kg). ****p < 0.0001. Arrowhead: autophagic compartments; asterisk: mitochondria. (Met metformin).

The number of multimembrane vesicular structures, consistent with autophagosomes, were significantly higher in ischemic retinas from mice treated with metformin 10 mg/kg (Fig. 3A, B).

**Fig. 4:**
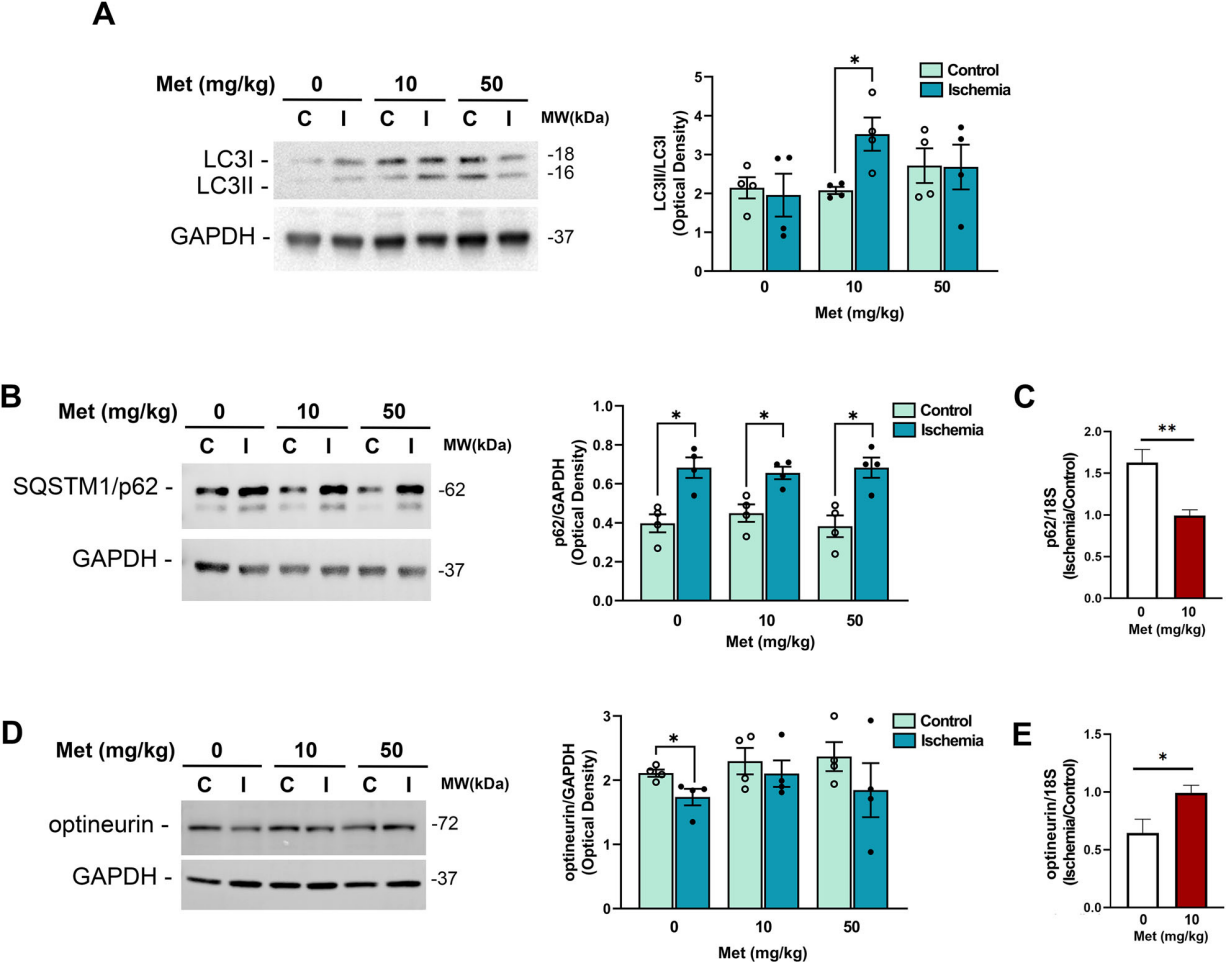
Metformin modulates the expression of autophagy-related proteins in retinas of mice subjected to ischemia/reperfusion injury. Representative immunoblots of LC3 (**A**), SQSTM1/p62 (**B**) and optineutin (**D**) in retinas from metformin- (10 and 50 mg/kg) or vehicle-treated mice subjected to unilateral ischemia (I); contralateral eye served as control (C). **A** A significant upregulation of LC3II/LC3I ratio was reported in ischemic retinas (I) from mice treated with metformin 10 mg/kg compared to control (C) (*p < 0.05). **B** Treatment with metformin (10 and 50 mg/kg) did not revert the accumulation of SQSTM1/p62 induced by ischemia (D) while prevented the decrease of optineurin. Histograms report the results of the densitometric analysis of the bands normalized to loading control (GAPDH). Animals were sacrificed at 24 h of reperfusion. Data are reported as mean ± s.e.m. (*n* = 4). *p < 0.05 (Met metformin, MW molecular weight). mRNA levels of SQSTM1/p62 (**C**) and optineurin (**E**) were assessed by qPCR in retinal samples from vehicle- or metformin-treated mice (10 mg/kg). Results were normalized to 18S rRNA and expressed as fold change of ischemic relative to control (*n* = 4) *p < 0.05; **p < 0.01.

Consistent with this observation, western blot analysis of LC3 (microtubule-associated protein 1 A/B light chain 3) showed a significant increase in the ratio between the autophagosome associated (LC3II) and the soluble form (LC3I) of the protein (LC3II/LC3I) in ischemic retinas from mice treated with 10 mg/kg metformin (Fig. 4A). No significant differences were observed in vehicle-treated or 50 mg/kg metformin groups (Fig. 4A).

### Metformin sustained PINK1/Parkin, optineurin-mediated, mitophagy in retina subjected to ischemia/reperfusion injury

SQSTM1/p62 and optineurin are autophagy receptors acting as molecular bridges between the ubiquitinated cargo and the autophagy machinery. While SQSTM1/p62 is primarily involved in macroautophagy [38, 39], optineurin is an essential mitophagy adaptor serving as autophagy receptor for damaged mitochondria [40, 41]. Indeed, its down-regulation has been associated with reduced mitophagy and accumulation of damaged organelles [41, 42].

In our previous study we observed an increase in SQSTM1/p62 expression in the ischemic retinas 24 h post-insult, suggesting a reduction of autophagy efficiency [23]. Metformin did not reduce the accumulation of SQSTM1/p62 in the ischemic retina, but prevented the transcriptional upregulation of *Sqstm1* mRNA observed in the vehicle-treated samples (Fig. 4B, C), suggesting that metformin may limit the stress response by mitigating tissue damage.

Under our experimental conditions, optineurin protein (Fig. 4D) and mRNA (Fig. 4E) levels were significantly reduced in the retina 24 h post-ischemia in vehicle-treated mice. This downregulation was prevented by metformin (10 mg/kg; Fig. 4D, E), suggesting preservation of mitophagy mechanisms.

**Fig. 5:**
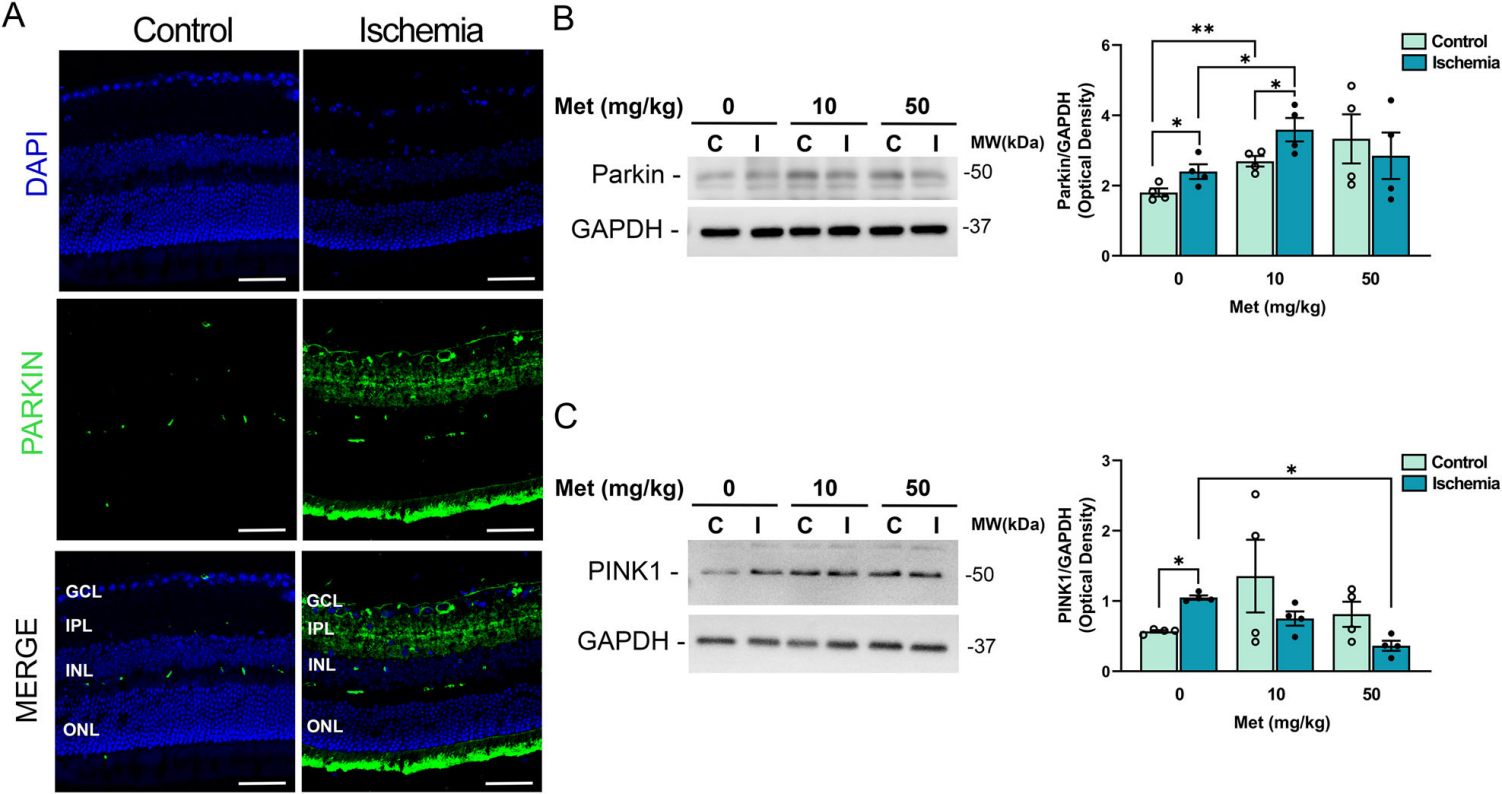
Metformin sustains PINK1/Parkin mitophagy in retina subjected to ischemia/reperfusion. **A** Confocal images showing Parkin immunoreactivity in retinas from control and ischemic eyes 24 h post-injury. Upregulation of Parkin was mostly localized at the innermost retinal layers (GCL and IPL). Nuclei were counterstained with DAPI (blue). Images are representative of three independent experiments. GCL ganglion cell layer, IPL inner plexiform layer, INL inner nucler layer, ONL outer nuclear layer. Scale bars 50 μm. **B**, **C** Western blotting of Parkin and PINK in control (C) and ischemic (I) retinas from mice treated with vehicle (0) or metformin (10 or 50 mg/kg). **B** Parkin was significantly upregulated 24 h post-ischemia compared to contralateral control. Treatment with metformin 10 mg/kg induced a further statistically significant increase of Parkin expression in both ischemic and contralateral retina as compared to the respective ones in the vehicle-treated group (0). No significant changes were detected in the retinas from mice treated with metformin 50 mg/kg. **C** Ischemia induced a significant upregulation of PINK1 expression compared to contralateral control retina in vehicle-treated mice (0). Treatment with metformin 10 and 50 mg/kg abrogated the differences in PINK1 expression between ischemic and contralateral control retinas. Histograms report the results of the densitometric analysis of the bands normalized to loading control (GAPDH). Data are shown as mean ± s.e.m. (*n* = 4). *p < 0.05; **p < 0.01 (Met metformin, MW molecular weight).

Parkin is an E3 ligase enzyme generating ubiquitin chains on the outer membrane of damaged mitochondria and recruiting and stabilizing optineurin to initiate autophagosome formation [41, 43, 44]. Parkin was significantly upregulated in the ischemic retina 24 h post-insult (Fig. 5A, B); immunoreactivity was mainly localized at ganglion cell layer (GCL) and inner plexiform layer (IPL) (Fig. 5A). Metformin 10 mg/kg further increased Parkin expression in both retinas subjected to ischemia and contralateral control compared to vehicle treated group (Fig. 5B). No effect was detected in the retinas from mice treated with metformin 50 mg/kg.

Parkin recruitments and activation to damaged mitochondria is mediated by the activity of the serine/threonine kinase PINK1 [44].

24 h after ischemia PINK1 expression was significantly upregulated in the ischemic retinas of vehicle treated group compared to contralateral control retina (Fig. 5). Treatment with both 10 and 50 mg/kg metformin abrogated the differences in PINK1 expression between ischemic retina and contralateral control (Fig. 5C).

### Metformin prevented the accumulation of LC3II and sustained Parkin expression in the optic nerve of mice subjected to retinal ischemia

Western blot analysis was performed on the optic nerve (ON) 24 h post-ischemia.

**Fig. 6:**
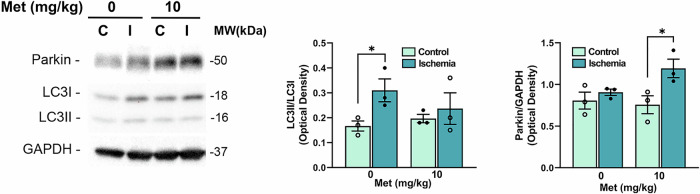
Metformin prevents LC3II accumulation and enhances Parkin expression in the optic nerve 24h-post retinal ischemia. Representative immunoblot of LC3 and Parkin in the optic nerves from mice treated with vehicle (0) or metformin (10 mg/kg) and subjected to unilateral ischemia (I). Contralateral eye was used as control (C). Metformin prevented LC3II accumulation and upregulated Parkin expression in the optic nerve from mice subjected to ischemic insult. Histograms show densitometric analysis of bands normalized to loading control (GAPDH). Data are reported as mean ± s.e.m. (*n* = 3). *p < 0.05 (Met metformin, MW molecular weight).

At variance with the results reported in the retina, LC3II accumulation reported in the ischemic ON from vehicle-treated mice, was prevented by metformin 10 mg/kg (Fig. 6).

Metformin treatment upregulated Parkin expression in the ischemic ON, supporting the hypothesis that mitophagy was sustained in both retina and ON by the treatment (Fig. 6).

### Comparative proteomic analysis of retinas from metformin treated mice

**Fig. 7:**
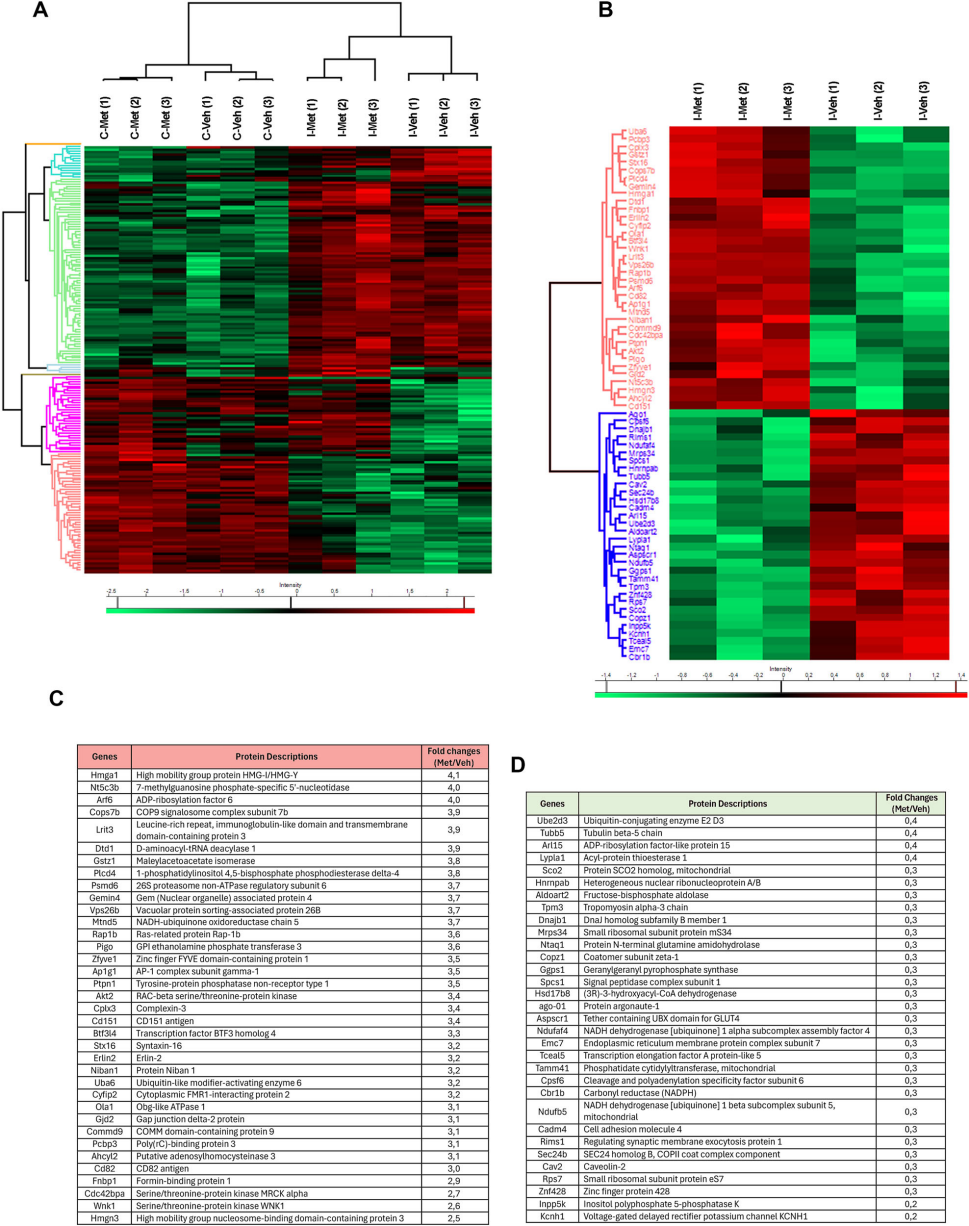
Proteomic profile of retinas from metformin-treated mice following ischemia/reperfusion injury. **A**, **B** Heatmaps of differentially expressed proteins in the retina from metformin- (Met) *vs*. vehicle (Veh)-treated mice subjected to unilateral ischemia (I). Contralateral eye was used as control (C). A total of 6032 proteins were identified and quantified. Statistical analysis was performed using one-way ANOVA (*p* < 0.01), identifying 182 proteins with significant differential expression between metformin- and vehicle-treated ischemic retinas. These proteins were grouped into five distinct clusters based on their abundance patterns. Tables of proteins significantly upregulated (**C**) and downregulated (**D**) by metformin treatment in ischemic-retinas.

Since metformin is a pleiotropic drug, to gain insight into the molecular mechanisms underpinning the observed neuroprotection, we performed comparative proteomic analysis of retinas from vehicle- and 10 mg/kg metformin-treated mice 24 h post-ischemia. A total of 6032 proteins were identified and analyzed via one-way ANOVA. The expression profile of the differentially expressed proteins was illustrated by a hierarchical cluster heatmap (Fig. 7A).

A statistically significance was reported for 182 proteins and five different clusters (and two single proteins) were identified based on protein abundance distributions (Fig. 7A).

Interestingly, several proteins included in the cluster indicated in pink color were upregulated in both ischemic and contralateral control retinas from metformin treated mice as compared to vehicle-treated (Fig. 7A). This suggest a change in retinal protein distribution which is independent of the ischemic insult and may be driven by either local or systemic effects of the drug. Sixty-eight proteins were found to be significantly different between metformin and vehicle treated ischemic retinas (Fig. 7B); among these, 36 were upregulated (Fig. 7C) and 32 were downregulated (Fig. 7D). Although pathway enrichment analysis yielded no statistically significant results, several relevant targets were identified.

Proteins involved in the formation of mitochondrial respiratory chain complexes (NADH-ubiquinone oxidoreductase chain 5, NADH dehydrogenase [ubiquinone] 1 alpha subcomplex assembly factor 4) and enzymes involved in the ubiquitination machinery (Ubiquitin-like modifier-activating enzyme 6, Ubiquitin-conjugating enzyme E2 D3) were reported as significantly modulated by the treatment (Fig. 7C, D).

ADP-ribosylation factor 6 (Arf6) was found to be one of the foremost upregulated proteins. Arf6 is an ADP ribosylation factor (Arf), member of the small G protein family, acting as a positive modulator of autophagy via regulation of phosphatidylinositol 4-phosphate 5-kinase (PIP5K) [45, 46].

Another notable upregulated protein was form-binding protein 1 (FNBP1) recently identified as a nutrient stress-responsive golgiphagy receptor. FNBP1 localizes at the fragmented Golgi membrane and engages LC3 thereby sequestering the fragmented Golgi membrane in autophagosomes and preventing metabolic stress in neurons [47].

Gene products involved in the trafficking of proteins between the trans-Golgi network and endosomes, such as AP-1 complex subunit gamma-1 (Ap1g1) and Vacuolar Protein Sorting-Associated Protein 26B (VPS26B), were also found to be significantly upregulated in the metformin-treated ischemic retina.

Conversely, inositol polyphosphate 5-phosphatase K (Inpp5k), which takes part in the lysosomal recycling pathway [48], was among the most significantly downregulated proteins in metformin-treated ischemic retina.

### Putative neuroprotective effect of metformin in diabetic patients with glaucoma

In a pilot retrospective clinical analysis, visual field (VF) changes (the gold standard in the diagnosis, monitoring, and management of glaucoma) were evaluated in diabetic patients with glaucoma treated with metformin (16 eyes of 8 patients) or insulin (16 eyes of 8 patients) over a six-month period.

The descriptive statistics for the parameters examined are presented in Tables 1 and 2.

**Table 1:** Descriptive statistics of normally distributed and categorical variables.

Parameter	Group	Number of Eyes	Male/Female (eyes)	Mean ± SD
Age	Metformin	16	8/8	71.50 ± 7.06
	Insulin	16	8/8	71.38 ± 7.10
Gender	Metformin	16	8/8	
	Insulin	16	8/8	
IOP T0	Metformin	16	8/8	15.19 ± 2.14
	Insulin	16	8/8	15.44 ± 2.22
IOP T6	Metformin	16	8/8	14.88 ± 2.25
	Insulin	16	8/8	15.00 ± 1.63

*SD* standard deviation, *IOP* intraocular pressure.

**Table 2:** Descriptive statistics of non-normally distributed variables.

Parameter	Group	Number of Eyes	Male/Female (eyes)	Minimum	Maximum	Median
MD T0	Metformin	16	8/8	−6.08	0.23	−0.98
	Insulin	16	8/8	−27	1.12	−1.09
MD T6	Metformin	16	8/8	−6.02	1.41	−0.63
	Insulin	16	8/8	−28.82	1.26	−1.94
PSD T0	Metformin	16	8/8	1.10	5.15	1.83
	Insulin	16	8/8	1/06	9.62	1.87
PSD T6	Metformin	16	8/8	1.06	5.97	1.86
	Insulin	16	8/8	0.99	8.51	1.73
VFI T0	Metformin	16	8/8	85	100	98.5
	Insulin	16	8/8	22	100	99
VFI T6	Metformin	16	8/8	88	100	99
	Insulin	16	8/8	13	100	98.5

*MD* mean deviation, *PSD* pattern standard deviation, *VFI* visual field index.

The Kolmogorov–Smirnov test indicated that age and IOP data were normally distributed, whereas VF parameters data were not; therefore, the paired or unpaired t-test was used for age and IOP comparisons and the Mann–Whitney U or Wilcoxon tests were applied for VF data, as appropriate. At T0, both groups were found to be comparable regarding age (*p* = 0.09) (metformin group 71.50 ± 7.06 years; insulin group: 71,38 ± 7.10 years), gender (*p* = 0.73), and glaucoma stage; as assessed by VF parameters including Mean Deviation (MD) (*p* = 0.75), Pattern Standard Deviation (PSD) (*p* = 0.93), and Visual Field Index (VFI) (*p* = 0.91). No intergroup differences emerged in terms of IOP at T0 and T6 (*p* = 0.75 and 0.86 respectively). No intragroup differences emerged from T0 to T6 (*p* = 0.40 and 0.31 in the metformin and insulin groups respectively); thus, ensuring that any differences observed during the study could more confidently be attributed to the treatment administered.

Over time, a notable distinction emerged between the two treatments: while patients in the insulin group experienced worsening of their visual function, those receiving metformin were stable.

**Fig. 8:**
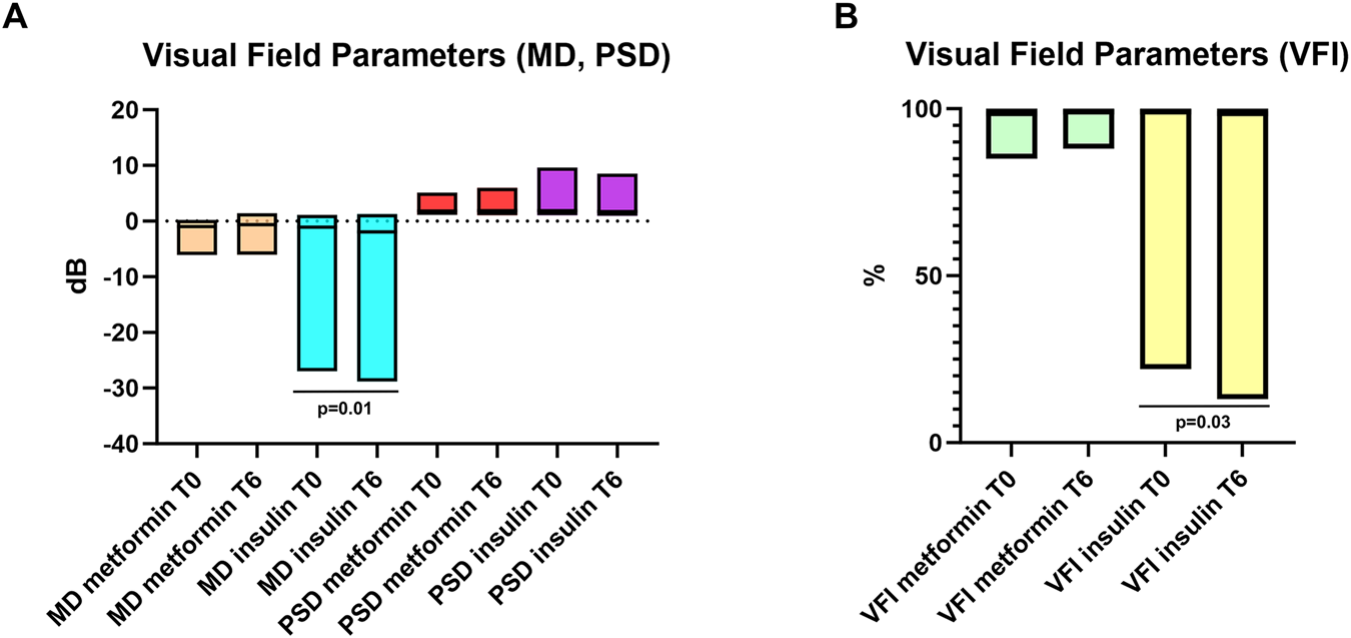
Effects of metformin and insulin treatments on visual field parameters in glaucoma patients. **A** Changes in Mean Deviation (MD) and Pattern Standard Deviation (PSD) from baseline (T0) to 6 months (T6) in patients treated with metformin or insulin. MD (beige/cyan bars) and PSD (red/purple bard) are shown. **B** Visual Field Index (VFI) changes from T0 to T6 in the same treatment groups. VFI values are expressed as percentage. Data suggest a differential impact of metformin and insulin on visual function over time.

After 6 months, MD values were not significantly modified in the metformin glaucoma group (*p* = 0.64). On the contrary, MD significantly worsened in the insulin glaucoma group (*p* = 0.01) at T6 (Fig. 8A). MD represents the average difference in sensitivity between the patient’s measured VF and that of an age-matched normative database. It is expressed in decibels (dB) and reflects the overall degree of VF loss. More negative MD values indicate greater global VF damage making the former parameter one of the most important to be evaluated in glaucoma management.

Furthermore, no significant differences were found in terms of PSD at T6 compared to T0 in the metformin and insulin groups (*p* = 0.70 and *p* = 0.89; respectively) (Fig. 8A). PSD quantifies localized irregularities in the VF by measuring the variability of individual test point deviations from the expected hill of vision. A higher PSD suggests greater localized VF defects, which are typical in early stages of glaucomatous damage.

Finally, when VFI was analyzed, values were not significantly modified in the metformin glaucoma group (*p* = 0.38), while there was a significant worsening in the insulin group (*p* = 0.03) comparing T0 to T6 (Fig. 8B). VFI is a weighted index expressed as a percentage, where 100% represents a normal VF and lower values indicate more severe loss. It places greater emphasis on the central VF and is less affected by cataract or diffuse loss, making it useful for tracking glaucoma progression.

The short observation time in this study possibly further reinforced our results highly reducing the risk that other factors (such as cataract) may have induced VF alterations.

## Discussion

Metformin, a widely prescribed drug for type 2 diabetes, is emerging as a promising neuroprotective agent, with its effects being investigated in a variety of neurodegenerative disorders [14, 49, 50]. In this study, we provide both preclinical and clinical evidence supporting the neuroprotective effect of metformin in the retinas of mice subjected to ischemia/reperfusion injury and its putative neuroprotective role in diabetic patients with glaucoma.

The mouse model is here used as an acute injury paradigm to investigate the molecular and cellular events contributing to RGC vulnerability and neuroprotection. The model has been widely used in preclinical studies and induces selective RGC loss without widespread photoreceptor degeneration [23, 51].

In this preclinical model of RGC degeneration, systemic administration of low-dose metformin effectively prevented RGC loss and preserved retinal morphology. Interestingly, higher dose of metformin failed to confer the same protection highlighting a non-linear dose- response. In agreement with our observation, in mice subjected to middle cerebral artery occlusion (MCAO), low doses of metformin (10 mg/kg) significantly reduced brain infarct size and prevented neuronal apoptosis, whereas higher doses (30-100 mg/kg) were not effective [32]. Similarly, in a rat model of global cerebral ischemia, low doses of metformin conferred the most significant protection to hippocampal CA1 neurons compared to higher doses [52].

This metformin fixed-dose effect has been described in several other experimental settings and for different outcomes [53, 54]. For instance, Wang et al. [55] reported that, in human neonatal macrophages, high doses of metformin enhanced pro-inflammatory M1 macrophage polarization, while low doses promoted anti-inflammatory M2 phenotypes [55]. Similarly, in hepatocytes, pharmacological doses of metformin increased mitochondrial respiration and fission, while supra-pharmacological concentrations reduced ATP levels and impaired mitochondrial activity [56].

Although the molecular mechanisms underlying metformin’s neuroprotective effects remain partially unravelled, AMPK activation is widely recognized as a central mediator [33, 57]. Nonetheless, growing evidence suggests that an optimal level of AMPK activation is critical for the metformin-mediated neuroprotection and excessive stimulation or off-target effects associated with higher doses may abrogate its beneficial actions [57–64].

In our study, we did not observe a significant different extent of AMPK phosphorylation between the neuroprotective and the ineffective doses, although we did not assess AMPK activity nor established its causal role in the observed protection. It is worth noting that the role of AMPK in neuroprotection is controversial [34]. Studies such as those by Vingtdeux et al. [65] have demonstrated that AMPK activation is crucial for neuroprotection, while others reported that excessive activity of AMPK might induce apoptosis under stress conditions [61]. Contradictory results were also found in animal models of glaucoma: Belforte et al. found that inhibition of AMPK in a mouse model of ocular hypertension led to RGC survival and restored axonal transport [66] and Harum-Or-Rashid and Inman (2018) reported that AMPK activation in DBA/2 J mice triggered a pro-inflammatory response [67]. Opposite results were reported by Brown and colleagues, showing that, in several preclinical models of retinal degeneration, AMPK activation promoted metabolic processes and prevented neuronal death [68].

In our study, the neuroprotection observed with the lower dose of metformin was accompanied by upregulation of the LC3II/LC3I ratio and, most importantly, the recurrence of multivesicular structures suggesting that metformin enhances the number of autophagosomes. The involvement of autophagy in the metformin-mediated neuroprotection reported in our model is also supported by the presence of the positive regulator of autophagy Arf6 [45] among the most upregulated proteins in the ischemic retinas from mice treated with metformin. Arf6 is an ADP ribosylation factor (Arf), member of the small G protein family, acting as a positive modulator of autophagy via its ability to regulate the formation of PIP2 through the stimulation of the phosphatidylinositol 4-phosphate 5-kinase (PIP5K) and regulating PLD (Phospholipase D) activity [45, 69].

Interestingly, at the protein level, we did not report any modulation of the autophagy receptor SQSTM/p62, suggesting that bulk autophagy flux is not enhanced by the treatment. *Viceversa*, the upregulation of optineurin and Parkin, key players in the PINK1/Parkin-mediated mitophagy pathway, and FNBP1, which has been identified as a nutrient stress-responsive golgiphagy receptor able to prevent metabolic stress in neurons [47], suggest that metformin may modulate selective forms of autophagy without broadly enhancing bulk autophagy.

The selective modulation of mitophagy-related proteins such as Parkin, PINK1, and optineurin support the hypothesis that mitochondrial quality control might be a central mediator of the neuroprotective effects reported in the retina. Overexpression of Parkin has previously been shown to promote RGC survival in a chronic hypertensive glaucoma rat [70], restore mitophagic flux, and enhance basal mitophagy [71, 72]. Accordingly, the central role played by mitochondria in metformin-mediated neuroprotection is supported by the findings of Zhang and colleagues who reported that metformin preserved mitochondrial membrane potential (MMP), reduced intracellular ROS generation, and induced mitochondrial fusion in a mouse model of retinal ischemia [73–75].

In the context of glaucoma, the pharmacological profile of metformin appears particularly promising.

In the preclinical part of the study, we employed a model that induces a reversible elevation of IOP, causing a hypoxic-ischemic insult with RGC loss, a hallmark of glaucoma. Importantly, the transient increase of IOP mimics the acute pressure spikes and neurodegenerative cascades observed in glaucomatous damage. Several studies have adopted these models to investigate neuroprotective strategies for glaucoma [23, 51, 76], reinforcing its validity in this translational context.

In the clinical part of the study, we investigated whether metformin could stabilize visual function in patients with glaucoma and coexisting diabetes. To minimize potential confounding factors, we specifically included glaucomatous patients with diabetes but without clinical evidence of diabetic retinopathy, thereby excluding those with overt microvascular complications. Additionally, both treatment groups (metformin- and insulin-treated) exhibited comparable glycaemic control and comorbidity profiles, further reducing the risk of bias attributable to diabetes-related factors.

Interestingly, in our pilot study, patients receiving insulin therapy exhibited a progressive decline in visual function, as measured by VF parameters. In contrast, those treated with metformin demonstrated stable visual performance over a six-month follow-up period. Although six months may appear relatively brief, this testing interval, previously employed in the Ocular Hypertension Treatment Study (OHTS), has been shown to be optimal for detecting disease progression in high-risk individuals [77, 78]. The significance of metformin’s role was previously underscored in a recent large-scale population-based study, which found that metformin use was associated with a 25% reduction in the risk of developing glaucoma This was also confirmed in a cohort study of 11,260 participants [28] and in a recent cross-sectional study that evaluated the relationship between metformin use and ocular structural and vascular parameters changes, specifically circumpapillary retinal nerve fibre layer (cpRNFL) thickness and whole image capillary density (wiCD), in patients with POAG or glaucoma suspect. Among 346 participants (577 eyes), 23% had diabetes, with the majority using metformin. Multivariable linear mixed-effect models, adjusting for relevant covariates, revealed that metformin use was significantly associated with increased wiCD and cpRNFL thickness. Additionally, longer duration of metformin use correlated with higher wiCD [79].

These findings suggest a potential neurovascular benefit of metformin in glaucoma, supporting our clinical results and warranting further longitudinal investigation to explore its therapeutic implications.

## Conclusions

Given its long-standing clinical use, favorable safety profile, affordability, and global accessibility, metformin emerges as a highly attractive candidate for repurposing in glaucoma management, particularly for patients who continue to experience disease progression despite well-controlled IOP.

However, the pleiotropic nature of metformin suggests that the observed neuroprotection likely involves multiple mechanisms that need to be further investigated. Moreover, systemic administration raises the possibility that some therapeutic effects may arise from peripheral, rather than direct retinal, actions.

We acknowledge that the current clinical evidence, being largely retrospective, is limited by potential confounding by indication, variability in treatment duration, and insufficient control for comorbidities. Therefore, rigorously designed, prospective randomized controlled trials are essential to validate metformin’s neuroprotective efficacy, define optimal dosing regimens and therapeutic windows, and elucidate the molecular pathways through which it preserves RGC integrity and visual function.

## Materials and methods

### Animals

Male C57BL/6 J mice (25–30 g) were purchased from Charles River (Lecco, Italy) and housed with a 12 h light–dark cycle with *ad libitum* access to food and water. Animal care and experimental procedures were carried out in accordance with the guidelines of the Italian Ministry of Health (D.L. 26/2014), the European Communities Council Directive (2010/63/UE) and the ARVO Statement for the Use of Animals in Ophthalmic and Vision Research. The experimental protocol was approved by the Italian Ministry of Health (Rome; NIH license no. 1026/2016-PR and no. 455/2023-PR). All surgical procedures were performed under deep anaesthesia and efforts were made to minimize the number of animals used and their suffering. The sample size (3–6 animals per group) was chosen based on previous studies using the same animal model [23, 25]. This number was sufficient in earlier work to detect a biologically relevant effect on RGC survival and functional recovery, and aligns with ARRIVE guidelines for exploratory preclinical studies.

### Retinal Ischemia

Retinal ischemia was induced in the right eye according to the method previously reported [23, 25]. Animals were deeply anesthetized by intraperitoneal injection (i.p.) of 15 mg/kg Xylazine (Sedaxilan®, Dechra Veterinary Products s.r.l., Turin, Italy) and 30 mg/kg Tiletamine-Zolazepam (Zoletil®, Virbac Srl, Milan, Italy) and laid on a heating pad to maintain the body temperature at 37 °C. Topical anaesthesia was induced by 0.4% Oxybuprocaine eye drops (Novesina®, Novartis Farma, VA, Italy). A 31-gauge infusion needle, connected to a 500 ml bottle of sterile saline, was inserted in the anterior chamber of the right eye, and the saline container was elevated to produce an intraocular pressure of 7080 mmHg for 60 min. IOP value (I, ischemic) was monitored by TonoLab (Icare, Finald). For each animal, the left eye was used as control (C, control). Body temperature was monitored and animals with values lower than 35.5 °C were excluded from the study. Mice were sacrificed, under deep anaesthesia, by cervical dislocation at 24 h or 7 days of reperfusion. To minimize the basal variations due to the circadian autophagy regulation [80], ischemia was induced between 8.00 am and 2.00 pm.

### Drug administration

Metformin hydrochloride (PHR1084, Sigma-Aldrich, Milan, Italy) was dissolved in sterile water for injection and stored at −20 °C. Metformin (10 or 50 mg/kg) or vehicle were injected i.p. once a day. Transient ocular hypertension was induced on the fourth day of treatment and animals were killed by cervical dislocation under deep anaesthesia after 24 h or 7 days. Animals were randomly allocated to either the untreated control group or the treatment group to minimize selection bias and ensure balanced group distribution.

### Protein extraction and western blotting

For western blot analysis, optic nerve and retina were quickly dissected, immediately frozen in dry ice, and stored at -80°C until use. Individual retinas or optic nerve were lysed in ice-cold RIPA buffer (50 mM Tris - HCl (pH 8), 150 mM NaCl, 1 mM ethylenediaminetetraacetic acid, 0.1% sodium dodecyl sulphate, 1% IGEPAL, 0.5% Sodium deoxycholate) containing protease (cod. P8349; Sigma-Aldrich, Milan, Italy) and phosphatase (cod. 524625, Calbiochem, La Jolla, CA, USA) inhibitor cocktails. Homogenization by tissue homogenizer lasted 40 s and lysates were kept on ice for 15 min and centrifuged for 15 min, 10,000 g at 4 °C. Supernatants were collected and assayed for protein concentration by the Bio-Rad DC Protein Assay Kit (Bio-Rad Laboratories, Milan, Italy). Equal amounts (10–15 µg) of total proteins were separated by SDS–polyacrylamide gel electrophoresis, transferred onto PVDF membranes (Immobilon-P, Sigma-Aldrich, Milan, Italy) and blocked with 5% non-fat milk in Tris-buffered saline (TBS) containing 0.05% Tween 20 (TBS-T) for 1 h at room temperature. Primary antibodies were prepared in 5% non-fat milk or 3% BSA in TBS-T and incubated overnight at 4 °C, followed by three washes with TBS-T and incubation with a species-specific horseradish peroxidase conjugated goat IgG as secondary antibody (Pierce Biotechnology, Rockford, IL, USA) for 1 h at room temperature. A list of the primary antibodies used is reported in Table 3. Protein bands were visualized by Western Blotting Luminol Reagent (Santa Cruz Biotechnology, Dallas, USA) and the chemiluminescence signal detected using X-ray films (Santa Cruz Biotechnology, Dallas, USA) or iBright 1500 (Thermo Fisher Scientific, Waltham, MA, USA). Bands were digitalized at 600 dpi and quantified using ImageJ software (NIH, Bethesda, MD, USA).

**Table 3:** Sources and dilutions of primary antibodies.

Target	Supplier and catalog no.	Dilution
LC3	MBL, PM036	1:2000
p62	Sigma, p0067	1:4000
Parkin	Santa Cruz, sc-32282	1:500
Optineurin	Proteintech, 10837-1-AP	1:4000
PINK1	Invitrogen, PA116604	1:1000
phospho-AMPK (Thr172)	Cell Signaling, 2535	1:1000
β-Actin	Sigma, A5441	1:1000
GAPDH	Applied Biosystem, AM4300	1:50,000

### Immunofluorescence

Eyes were enucleated and fixed in 4% paraformaldehyde at 4 °C for 2 h, the anterior cup and the lens removed, and the posterior cup cryopreserved in increasing concentrations of sucrose as follows: 10% sucrose for 1 h, 20% sucrose for 2 h, 30% sucrose overnight. Specimens were included in Optimal Cutting Temperature compound (Tissue-Tek®, Sakura Finetek Europe, The Netherlands) and frozen. 14 μm thick sections were cut with Cryostat (Leica), mounted onto Superfrost Plus glass slides (Thermo Fisher Scientific, Waltham, MA, USA) and stored at −20 °C until use. For cellular and subcellular localization of specific antigens, retinal sections were thawed, air-dried, post-fixed in 4% paraformaldehyde for 10 min and washed in 0.1 M PBS (pH 7.4). Sections were permeabilized with 0.3% Triton-X100 (Sigma-Aldrich, Milan, Italy) for 1 h, washed three times with PBS and blocked with 10% donkey serum (Sigma-Aldrich, Milan, Italy) at room temperature for 1 h. Slides were incubated with the anti-Parkin antibody (Santa Cruz, sc-32282; 1:50) in 5% donkey serum overnight, washed with PBS-0.1% Tween 20 and incubated with anti-mouse Alexa Fluor 488 (Molecular Probes, Eugene, OR, USA) at room temperature for 1 h (1:1250). Sections were mounted with Vectashield mounting media with DAPI (Vector Laboratories, Burlingame, CA, USA) and images acquired using a confocal microscope (Olympus FV3000, Tokyo, Japan).

### PCR

Total RNA was extracted with Triazol (Invitrogen, Carlsbad, CA). One microgram of total RNA was reverse transcribed in a final volume of 50 μl using the High Capacity cDNA Reverse Transcription Kit (Thermo Fisher Scientific, Waltham, MA, USA); cDNA was diluted 1:3 in nuclease free water.

Quantitative PCR was performed using the following primer sequences:

- Specific primers for optineurin

Forward sequence 5’-CCAGCAGACTTACCTGTTTC-3’

Reverse sequence 5’- GCAGGAGTGAATCGGAATAC-3’

- Specific primers for p62

Forward sequence 5’- CACCAGAAGATCCCAATGTC-3’

Reverse sequence 5’- CCTCCATGTTCCACATCAAT-3’

- Specific primers for 18S

Forward sequence 5’-C GGCGACGACCCATTCGAAC-3’

Reverse sequence 5’- GAATCGAACCCTGATTCCCCGTC-3’

PCR reactions were performed in the QuantStudioTm 3 Real Time PCR System (Thermo Fisher Scientific, Waltham, MA, USA) using 0.15 μmol/L of each primer. PowerUp™ SYBR™ Green MasterMix (Thermo Fisher Scientific, Waltham, MA, USA) with the dissociation protocol was used for gene amplification. Each sample was normalized to its 18S rRNA (18S) content. Results were expressed as n-fold differences relative to a calibrator and calculated using the ΔΔCt method.

### Light and transmission electron microscopy

Mice were sacrificed by cervical dislocation 24 h following retinal ischemia. Eyes were enucleated, fixed for 20 min in 4% PFA at 4 °C and the anterior cup and lens removed. Posterior cup was fixed in Karnowsky fixative (4% paraformaldehyde and 2.5% glutaraldehyde in 0.1 M phosphate buffer, pH 7.4), and then postfixed with 1% osmium tetroxide in 0.1 M phosphate buffer for 1 h at room temperature. After standard ethanol dehydration, samples were embedded in an Epon-Araldite 812 mixture. Sections were obtained with a Reichert Ultracut S ultramicrotome (Leica, Nussolch, Germany). Semi-thin sections (600-nm-thick) were stained with crystal violet and basic fuchsin and then observed with an Eclipse Ni-U microscope (Nikon, Tokyo, Japan) equipped with a DS-SM-L1 digital camera (Nikon). Ultra-thin sections (70-nm-thick) were stained with uranyl acetate and lead citrate, and then observed with a JEM-1400Flash transmission electron microscope (TEM) (Jeol, Tokyo, Japan). TEM images were acquired with a Morada digital camera.

A morphometric analysis was performed on electron micrographs with Fiji (ImageJ, NIH, Bethesda, MD, USA). For each treatment, six TEM images were taken at 12,000x magnification; for each micrograph, a cytoplasmic area of 100 μm^2^ was selected in the ganglion cell layer (GCL) and the number of autophagic compartments was counted.

### Retrograde labeling of RGCs

RGCs were retrogradely labeled by stereotaxically injecting the fluorescent tracer FluoroGold (FG; Fluka, Sigma-Aldrich, Milan, Italy) into the superior colliculi. Four days after the ischemic insult, mice were deeply anaesthetized, immobilized in a stereotaxic device (Kopf 900, Analytical Control, Milan, Italy) and the positions of superior colliculi were identified by the stereotaxic coordinates reported in Paxinos and Franklin Mouse Brain Atlas (2019). The skull was exposed and 2 μl of 2% FG solution were injected on both sides of the skull 4 mm posterior to the bregma, 1 mm lateral to the sagittal suture and 1.6 mm ventral from the bone surface using a Hamilton Neuros-syringe with a 33-gauge needle (Hamilton Europe, Bonaduz, Switzerland). The skin was then sutured, and a 0.3% tobramycin ointment was applied (Tobral®, Alcon, Milan, Italy). Animals were killed 7 days after ischemia and eyeballs enucleated and fixed for 20 min in paraformaldehyde 4% in PBS. The anterior segment of the eye was removed and the posterior eyecup additionally fixed for 30 min. Isolated retinas were divided into four quadrants (superior, inferior, nasal and temporal) and mounted on slides using the ProLong Gold Antifade Mountant (Thermo Fisher Scientific, Waltham, MA, USA). Thirty-two images *per* retina (three from the peripheral, three from the middle, and two from the central retina for each quadrant) were acquired using a confocal microscope (Olympus FV3000, Tokyo, Japan) and subjected to cell count using ImageJ software (NIH, Bethesda, MD, USA). The total number of labeled cells in the eye subjected to retinal ischemia was compared with contralateral and expressed as percentage of RGC survival.

### Proteomic analysis

Proteins were digested according to the Protein Aggregation Capture (PAC) protocol [81]. Briefly, protein solutions were diluted in RIPA buffer to a volume of 30 ml (15 µg of total proteins). Reduction and cysteine alkylation proceeded by adding 4.5 ml of 100 mM DTT dissolved in Tris HCl pH 8.5 (1 h at 37 °C) and 3.6 ml of 200 mM iodoacetamide (1 h at 37 °C). 19.05 ml of the protein solution (10 mg) were processed by the PAC protocol. MagReSyn® Hydroxyl microparticles (Resyn Biosciences) were equilibrated twice in 70% acetonitrile, then resuspended in 70% acetonitrile in order to obtain the initial storage volume of the beads. Five µl of beads suspension were utilized per sample and placed in a 2 ml Eppendorf tube. Reduced and alkylated proteins (19.05 ml) were added to the beads. Then, 45 µl of acetonitrile were added to initiate protein precipitation. The solution was pipetted vigorously for 20 times and then incubated at 1100 rpm for 10 min. Beads were then separated using a magnet for a few seconds. The supernatant was removed, and beads were washed twice with 200 µl of 100% acetonitrile and one with 200 µl of 70% ethanol while still in the magnetic rack. The tubes were then removed from the magnetic rack. Trypsin solution (50 µl of 50 mM TEAB + 1 µl of trypsin concentrated 0.2 µg/µl) was added and digestion was allowed to proceed overnight at 37 °C and 1100 rpm. The following day, the supernatants containing the peptides were removed, and magnetic beads were washed with 50 µl of 0.1% FA (v/v). The two supernatants were pooled and the peptide mixtures were directly injected for nanoLC-MS/MS analysis. NanoLC-MS/MS analysis was performed on an EASY1200 LC system coupled to an Orbitrap Exploris 480 mass spectrometer (Thermo Fisher). Peptides were separated using an in-house made 15 cm analytical column packed with 3 μm-C18 silica particles (Dr. Maisch) essentially as described in Prestagiacomo et al. [82]. Briefly, gradient elution of peptides was achieved at 300 nl/min using a 140 min gradient (from 3% B to 8% B in 40 min, then from 8% B to 32% B in 120 min, followed by column regeneration at 100% B for 20 min). Mobile phase A was 0.1% formic acid containing 2% acetonitrile, whereas mobile phase B was 0.1% formic acid containing 80% acetonitrile. The nanoLC effluent was directly electrosprayed into the mass spectrometer in positive ion mode (1800 V). The mass spectrometer operated in DIA mode, using 60 sequential acquisition windows covering an m/z range of 350–1000. Full MS scans (350–1000 m/z) at 60,000 resolution were followed by 60 sequential MS/MS windows: 45 scans in the range 350–710 having 8 m/z width, 9 scans in the range 710–870 having 16 m/z width, 5 scans in the range 870–1010 having 28 m/z width. In all cases, window overlap was 0.5 m/z, target was 500% (5e5), normalized collision energy was 25, resolution was 30,000, maximum injection time was 50 ms. For the data analysis, library generation was achieved in Spectronaut Pulsar (Biognosys, v.16.0) using default parameters. Details can be found in Prestagiacomo et al. [82]. MS/MS spectra were searched against the *Mus musculus* protein database accessed on May 23th 2022 (55.315 sequences). DIA data analysis was performed on the same platform (Spectronaut) using default parameters. The “Major group top N” parameter (number of peptides used for quantification) was between 1 and 10, data were imputed using a global imputation strategy and automatic normalization was adopted in Spectronaut. For statistical analysis of Spectronaut output, Perseus software was used as follows: the Protein Group Quantity from the Spectronaut analysis was imported. Data were transformed in logarithmic scale (log2). After filtering (at last two valid LFQ values in at least one group), two-sample t test has been used to assess the statistical significance of protein abundances using a 5% permutation-based FDR adjustment. A scaling factor was used for corrections 0:0.2.

### Clinical study

For this pilot retrospective study, clinical notes of glaucomatous diabetic patients of the glaucoma department of the University Hospital of Rome Tor Vergata were reviewed.

All methods were carried out in accordance with the relevant guidelines and regulations, including the principles of the Declaration of Helsinki. Ethical approval for this study was obtained from the Independent Ethics Committee of University Hospital “Tor Vergata”, Rome, Italy (Protocol name: INSUMET, Protocol number: 105.22). Informed consent was obtained from all participants involved in the study. All data were fully anonymized prior to analysis, and no images or other materials are included that could lead to the identification of individual participants.

Two groups of patients with glaucoma and diabetes, homogeneous in terms of age and gender, were extrapolated. The study was approved by the internal review board of the University Hospital of Rome “Tor Vergata”.

The first group (metformin glaucoma group) included patients aged between 18 and 90 years. They must have a concomitant diagnosis of open-angle glaucoma and type 2 diabetes without signs of diabetic retinopathy; they must be under glaucoma treatment with IOP lowering medications and must be under glucose-lowering treatment with metformin. The clinical record must also report at least two 24-2 visual field (VF) carried out 6 months apart from each other.

The second group (insulin glaucoma group) included patients aged between 18 and 90 years. They must have a concomitant diagnosis of open-angle glaucoma and type 2 diabetes without signs of diabetic retinopathy; they must be under glaucoma treatment with IOP lowering medications and must be under glucose-lowering treatment with insulin. Metformin use was an exclusion criterion. The clinical record must also report at least two 24-2 VF carried out 6 months apart from each other.

Patients treated with brimonidine, citicoline, and coenzyme Q10 or affected by other neurological disorders were excluded.

The following data have been collected from these groups:

- Age
- Gender
- Type of glaucoma
- Topical and systemic therapy for glaucoma at the time of the exams (type of drugs and dosage)
- IOP at baseline (T0) and after 6 months (T6)
- 24-2 VF exam with particular attention to mean deviation (MD), pattern standard deviation (PSD), and visual field index (VFI).

### Statistical analysis

#### Data from Animal study

Data were expressed as mean ± standard error of the indicated number of independent experiments and evaluated statistically for difference by ANOVA analysis of variance followed by Tukey–Kramer test for multiple comparisons. Where indicated, Student’s *t* test was used to evaluate differences between two means. A value of *p* ≤ 0.05 was considered significant. The statistical significance was analyzed using the GraphPad Prism 8.3.0 software (GraphPad Soft-ware, Inc., San Diego, CA, USA).

#### Data from human study

All data have been entered into a GraphPad Prism 8.0.1 database (GraphPad Software 2365 Northside Dr. Suite 560 San Diego, CA 92108) and the statistical analysis has been performed using the same software. For all variables, the Gaussian (Normal) distribution has been verified using the Kolmogorov–Smirnov test. Parameters with Gaussian distribution have been described with the mean ± standard deviation, alternatively, they have been described with median (minimum; maximum). For the comparison of normally distributed variables, paired or unpaired t-tests were applied as appropriate. In cases involving non-normally distributed variables, non-parametric tests such as the Mann–Whitney U test and the Wilcoxon signed-rank test were utilized, as deemed suitable. For comparisons of categorical variables (frequencies), the Chi-square test and/or Fisher’s exact test were applied as appropriate. A value of *p* ≤ 0.05 is considered statistically significant.

## Supplementary information

UNCROPPED WESTERN BLOTS

## Data Availability

All data generated or analyzed during this study are available from the corresponding author upon request.
