# Supplementary material for: Metformin protects retinal ganglion cells in a preclinical model of retinal ischemia/reperfusion injury and stabilizes visual field in diabetic patients with glaucoma
Source: Cell Death Discov. 2025 Nov 24;11:546. doi: 10.1038/s41420-025-02824-y (PMC12645046; doi:10.1038/s41420-025-02824-y)

Western Blotting Figure 1 D

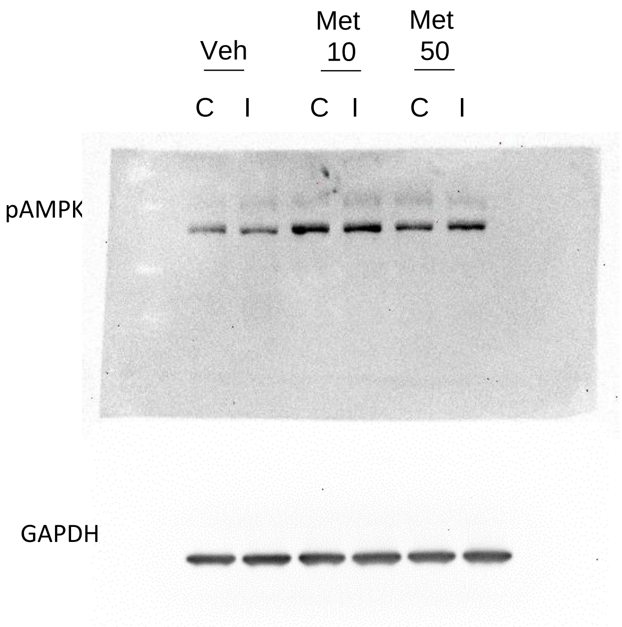

Western Blotting Figure 4 A

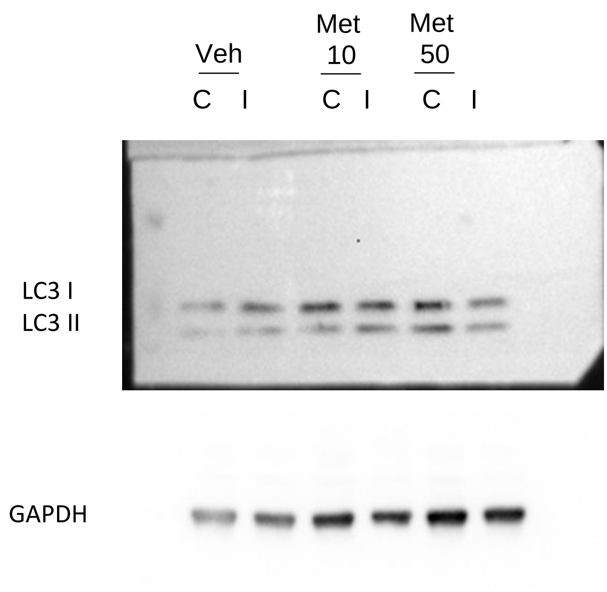

Western Blotting Figure 4 B

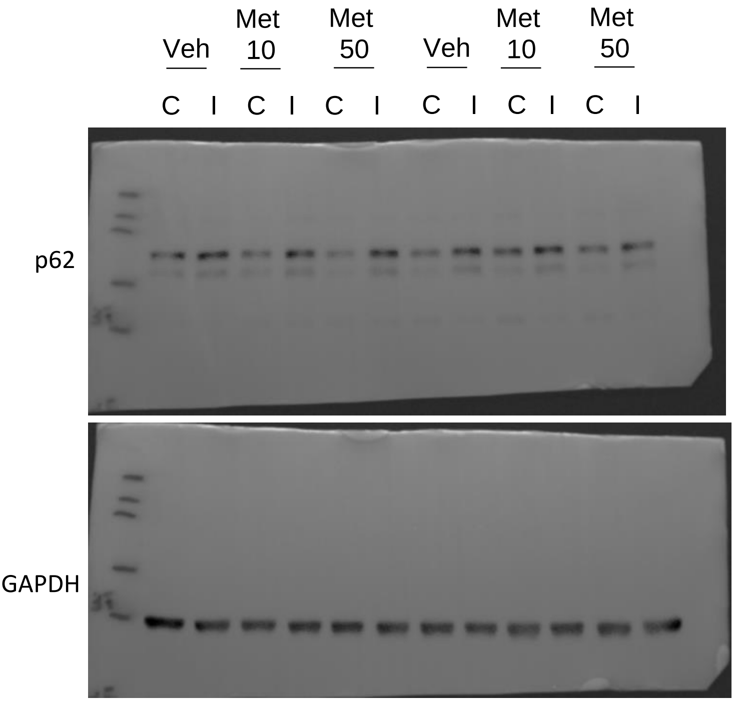

Western Blotting Figure 4 D

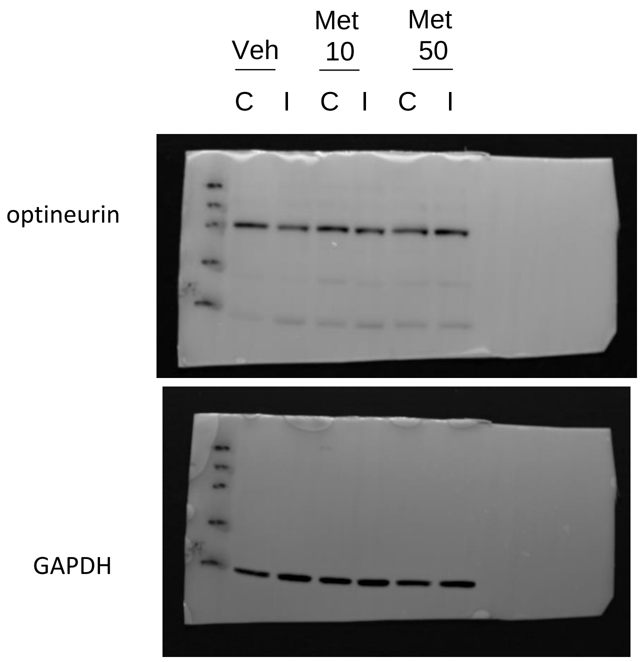

Western Blotting Figure 5 B

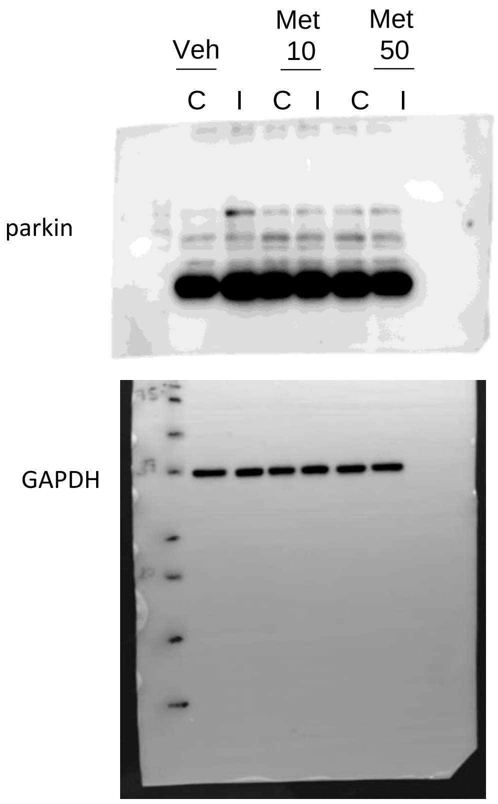

Western Blotting Figure 5 C

Veh
Met 10
Met 50

C I C I C I

PINK

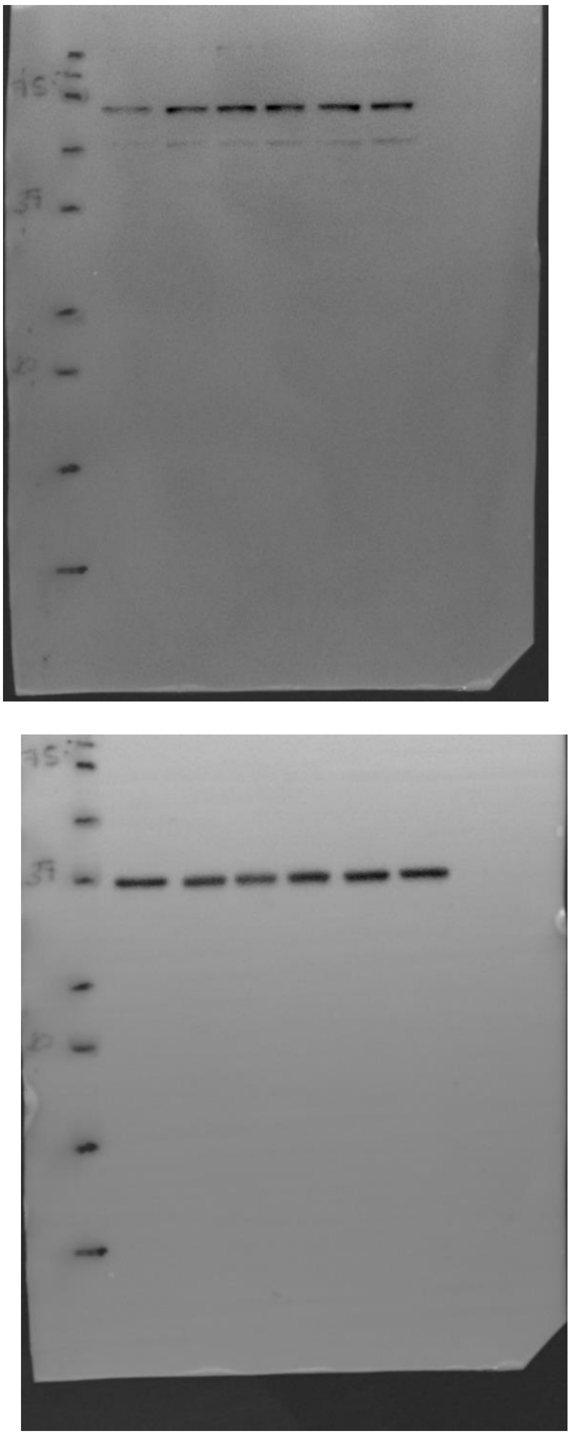

GAPDH

ON: Western Blotting Figure 6

Veh
Met 10

C I C I

LC3 I
LC3 II

parkin

GAPDH

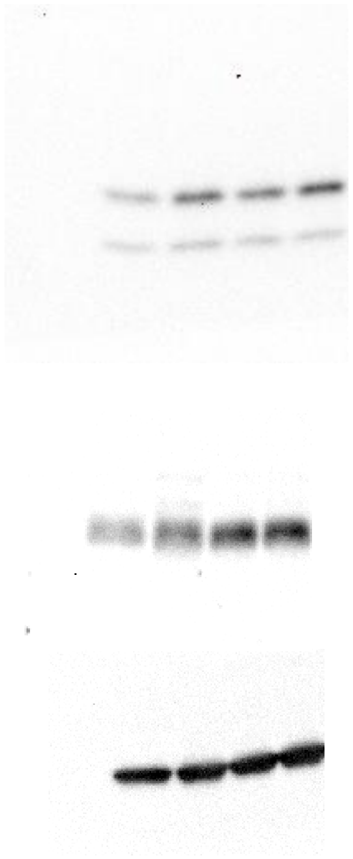

Supplement: Supplementary file 1 — UNCROPPED WESTERN BLOTS [file 41420_2025_2824_MOESM1_ESM.pdf]
